# Subchondral bone repair potential of an osteochondral scaffold augmented with BMP-2 or strontium-enriched amorphous calcium phosphate: a co-culture *in vitro* model

**DOI:** 10.3389/fmed.2026.1738671

**Published:** 2026-02-11

**Authors:** Manuela Salerno, Marta Columbaro, Stefania Pagani, Janis Locs, Jana Vecstaudza, Laura Dolcini, Milena Fini, Gianluca Giavaresi, Giuseppe Filardo

**Affiliations:** 1 Applied and Translational Research Center, IRCCS Istituto Ortopedico Rizzoli, Bologna, Italy; 2 Electron Microscopy Platform, IRCCS Istituto Ortopedico Rizzoli, Bologna, Italy; 3 Surgical Sciences and Technologies, IRCCS Istituto Ortopedico Rizzoli, Bologna, Italy; 4 Faculty of Natural Sciences and Technology, Institute of Biomaterials and Bioengineering, Riga Technical University, Riga, Latvia; 5 Baltic Biomaterials Centre of Excellence, Headquarters at Riga Technical University, Riga, Latvia; 6 Fin-Ceramica Faenza S.p.A, Faenza, Italy; 7 Scientific Direction, IRCCS Istituto Ortopedico Rizzoli, Bologna, Italy; 8 Faculty of Biomedical Sciences, Università della Svizzera Italiana, Lugano, Switzerland

**Keywords:** BMP-2, calcium phosphates, mesenchymal stromal cells, osteochondral defect, scaffold

## Abstract

**Purpose:**

A collagen/collagen-magnesium-hydroxyapatite (Col/Col-Mg-HA) scaffold is currently used in the clinical practice to address osteochondral lesions (OCL). However, suboptimal bone regeneration still limits its overall reparative potential. The aim of this study was to test *in vitro* the osteoinductive potential of two different augmentation strategies: the addition of Bone Morphogenetic Protein-2 (BMP-2) or the incorporation of strontium ions-enriched amorphous calcium phosphate (Sr-ACP) granules.

**Methods:**

Human mesenchymal stromal cells (MSCs) were seeded on the differently modified scaffolds and unmodified material used as a control, and grown for 14 days in a co-culture system in the presence of primary osteoclasts and conditioned medium of endothelial cells. The potential of the BMP-2- and Sr-ACP-enriched scaffolds compared to the control was then evaluated in terms of MSCs adhesion and morphology, metabolic activity, osteogenic differentiation, and osteoclasts’ markers expression.

**Results:**

Morphologically, both modified scaffolds sustained good cell adhesion. More secreted matrix was observed on the BMP-2 scaffold, together with higher metabolic activity and an upregulation of most of the evaluated osteoblast-related genes compared to the control and the Sr-ACP scaffold. Conversely, in the presence of the Sr-ACP scaffold, lower metabolic activity and a slower activation of osteoblast-related markers, together with a tendency to stimulate the osteoclasts’ activity, was observed.

**Conclusion:**

Overall, both augmentation strategies were able to favor the adhesion and growth of MSCs compared to the unmodified scaffold, with the BMP-2-modified scaffold promoting more the differentiation of MSCs toward a mature osteoblastic phenotype than the Sr-ACP-modified scaffold, and the latter promoting more osteoclast activation.

## 1 Introduction

Structural damage of the joint consequent to trauma or disease may lead to the development of osteochondral lesions (OCL), which involve the entire osteochondral unit (1). Cartilage and subchondral bone are characterized by different intrinsic biological and biomechanical properties and present a limited self-healing ability (2). An optimal regeneration of both tissues is paramount to achieve an adequate restoration of the osteochondral unit and, subsequently, of articular function. The presence of a healthy articular cartilage and an intact subchondral bone tissue ensures correct weight distribution, joint lubrication, and mechanical support during movement (3). If not properly treated, these defects may progress to osteoarthritis (OA), ultimately requiring joint replacement surgeries (4–6) with a consequent important social and economic burden (7, 8).

One of the most used strategies for the clinical management of OCL is a biomimetic material made of a collagen-based organic phase and a collagen comprising magnesium substituted-hydroxyapatite (Mg-HA) mineral phase (Col/Col-Mg-HA). This scaffold is a chemically and morphologically graded device, with three layers consisting of different ratios of collagen and HA resembling the composition of the extracellular matrix of articular cartilage, tidemark zone, and subchondral bone tissue, and Mg introduced to increase the physicochemical, structural, and morphological affinities with natural bone (9). The scaffold demonstrated biocompatibility, resorbability, and flexibility (10, 11), with its hydrophilic porous structure favoring the recruitment of progenitor cells from the bone marrow surrounding the implantation site (12), thus supporting and stimulating chondrogenesis and osteogenesis processes (13, 14). In the clinical practice, this scaffold showed a good stability and relevant knee function improvement up to 2- and 10-year follow-ups (5, 15). However, these good results in terms of cartilage restoration and clinical outcome are in contrast with signal alterations persisting over time on MRI scans indicating a limited subchondral bone repair (5, 12). To improve the repair potential of the implant’s subchondral bone layer, two alternative strategies have been proposed: the adsorption of the bone morphogenetic protein 2 (BMP-2) or the addition of amorphous calcium phosphate with Strontium (Sr) ions (Sr-ACP), which showed promising preclinical results in terms of bone regeneration (16–18).

Exploring the effects of these approaches on the behavior of cells involved in subchondral bone homeostasis may provide a better comprehension of the mechanisms underlying the cell/scaffold interaction and of the scaffolds’ osteogenic potential. For this purpose, the same implemented biomaterials were recently observed in two different *in vitro* models. One simulated the aggressive environment representative of aged and osteoarthritic joints, by seeding normal human osteoblasts (NHOsts) on the scaffolds in presence of inflammatory mediators and reactive oxygen species. In this *in vitro* set up, developed in a very short-term culture time, the BMP-2 addition was more effective than the Sr-ACP-modified in sustaining the activity of bone-forming cells (16). Subsequently, another study was set up at longer culture time with NHOst in a co-culture system, to mimic the physiological environment of the subchondral bone, a complex metabolically active tissue populated by different cell types (19). Co-culture systems are recognized as value-added models in joint regenerative medicine and osteoarthritis (OA) research (20, 21), as they promote cell–cell interactions through paracrine signaling compared with single cultures (22). Accordingly, in this second model, osteoblasts and osteoclasts—central to bone remodeling—were co-cultured with endothelial cell conditioned medium, serving as a surrogate for the bone vasculature and providing soluble factors that can stimulate osteoblast activity (23, 24).

Based on the two studies described above, this research further deepened the understanding of cellular responses to these functionalized scaffolds by assessing gene expression and ultrastructural morphology. For this purpose, the tricultural approach was used once again, replacing the osteoblasts with MSCs. Thanks to the complexity and characteristics of this system, it can be used as a valuable screening tool for selecting biomaterials that should undergo *in vivo* preclinical and clinical testing. The *in vitro* observation of all the main cell players involved in the bone regeneration process can provide an indication of their in vivo behavior, significantly helping to select more effective biomaterials in a translational way.

## 2 Materials and methods

### 2.1 Scaffolds

A material composed of 60% equine (type I) collagen/40% collagen -magnesium-hydroxyapatite (Col/Col-Mg-HA) (Maioregen, Fin-Ceramica, Faenza, Italy), mimicking the composition of the subchondral bone and having a thickness of 2 mm and a diameter of 8 mm was used in the present study as osteochondral control scaffold (OC). BMP-2-modified scaffolds (OC + BMP-2) were obtained by the adsorption of BMP-2 (R&D System, Minneapolis, MN, United States) to the control material. Briefly, 4 μg BMP-2 were dissolved in a few microliters and gently added dropwise on the top surface of each dry scaffold, to enable even distribution and prevent dispersion. Scaffolds were then incubated for 30 min at 37 °C just before cell seeding. Sr-ACP-modified scaffolds (OC + Sr-ACP) were obtained by addition of 100–150 μm sized Sr-ACP granules to the scaffold’s Col/Col-Mg-HA layer (30 w/w%). Both scaffolds were previously characterized and investigated by Xu et al. (17, 18).

### 2.2 Cell cultures

Human bone marrow-derived MSCs were purchased from the American Type Culture Collection (ATCC, Rockville, Maryland, United States) and expanded in appropriate growth medium (GM) (MesenCult Human Basal Medium, Stem Cell Tech, VODEN, Italy) supplemented with 10% fetal bovine serum (FBS) (LONZA, Walkersville, MD, United States). MSCs at passage 5 were used.

Human umbilical vein endothelial cells (HUVEC, LONZA) were cultured in commercial endothelial growth medium (EBM-2 Endothelial cell basal medium-2) supplemented with 2% FBS, human Vascular Endothelial Growth Factor (hVEGF), human Epidermal Growth Factor (hEGF), human Fibroblast Growth Factor-Basic (hFGF-B), R3-Insulin-like Growth Factor (R3-IGF), Hydrocortisone, Heparin, Gentamicin, Ascorbic Acid (EGM™-2, LONZA). Conditioned medium from HUVEC at confluence was collected after a 24-h starvation period, centrifuged to eliminate cell debries, and immediately used for the co-culture experiments.

Human osteoclasts were obtained from Peripheral Blood Mononuclear Cells (PBMCs) derived from venous blood of healthy human adult male donors after written informed consent (Ethics Committee—CE AVEC—Approval no. 191/2019/Sper/IOR, 04/19). For each experiment, PBMCs from different donors were used. The inclusion and exclusion criteria defined in the clinical study were aimed at minimizing biological differences among donors.

PMBCs were isolated on a Ficoll-Hystopaque gradient (Sigma Aldrich, St. Louis, MO, United States) according to the manufacturer’s instructions and seeded at the density of 1 × 10^6^ cells/cm^2^ in Dulbecco’s modified Eagle medium (DMEM high glucose, Sigma-Aldrich) supplemented with 10% FBS. After 24 h, non-adherent cells were discarded and the growth medium was supplemented with 25 ng/mL of macrophage colony-stimulating factor (M-CSF), 30 ng/mL of Receptor activator of nuclear factor kappa-*Β* ligand (RANKL), and 10^−7^ M of parathyroid hormone (PTH) (Peprotech, Rocky Hill, NJ, United States) (osteoclasts differentiation medium). After 7 days, the osteoclast morphology was assessed by Tartrate-Resistant Acid Phosphatase-Positive (TRAP, Sigma-Aldrich) staining, according to the manufacturer’s instructions. Only differentiated osteoclasts were used for the co-culture experiments. All the cultures were maintained at 37 °C in a 5% CO_2_/95% air-controlled atmosphere.

### 2.3 Co-culture model

A co-cultured model was established using Transwell polycarbonate membrane cell culture inserts on a 12-well plate (Thermo Fisher Scientific, Waltham, MA, United States). Osteoclasts were first seeded in the bottom well and were allowed to differentiate for 1 week, as described in the previous paragraph. Then, the scaffolds (OC, OC + BMP-2, and OC + Sr-ACP) were placed in the Transwell insert and preconditioned with 50 μL of GM for 1 h at 37 °C. The MSCs (2×10^5^ cells/scaffold) were then seeded dropwise on the scaffold surface and let to adhere for 2 h at 37 °C. Finally, a culture medium with the following composition was added: 40% of MSCs differentiation medium, composed of GM supplemented with 50 μg/mL of ascorbic acid, 7 mM of *β*-glycerophosphate, and 1×10^−7^ M dexamethasone (all from Sigma Aldrich) + 40% osteoclast differentiation medium + 20% HUVEC conditioned medium. The medium, a mixture of each specific culture medium related to cell types and proportional to the respective cell density (25) was replaced twice a week. After 7 and/or 14 days, the cultures were evaluated for cell morphology, metabolic activity, gene expression, and matrix synthesis. The experiment was repeated three times using different cell batches.

### 2.4 Transmission electron microscopy (TEM) analysis

At the end of the experiment (i.e., 14 days), MSCs cultured on the scaffolds were fixed with 2.5% glutaraldehyde in 0.1 M cacodylate buffer (Sigma-Aldrich) for 1 h at room temperature followed by 3 h at 4 °C. Afterwards, the samples were washed with 0.1 M cacodylate buffer, post-fixed with osmium tetroxide (Electron Microscopy Sciences, Hatfield, PA, United States) for 2 h, dehydrated in graded concentrations of ethanol and propylene oxide (Sigma-Aldrich), and finally embedded in EPON 812 (Electron Microscopy Sciences). Ultrathin cross sections (80 nm) were stained with uranyl acetate (Electron Microscopy Sciences) and lead citrate (Fluka Honeywell, NC, United States) and observed with a Jeol Jem-1011 electron microscope at 100 kV (Jeol LTD, Tokyo, Japan). Images were captured using an Olympus digital camera (Morada CCD camera, Olympus-Soft Imaging Solution GmbH, Münster, Germany) and iTEM software (Software: OSIS model iTEM, Olympus).

### 2.5 Alamar Blue assay

Cell metabolic activity of MSCs grown on the scaffolds was quantified after 7 and 14 days of culture by the Alamar Blue assay (Thermo Fisher Scientific). Briefly, the MSCs/scaffolds constructs were transferred to a new sterile plate to avoid interferences due to the presence of osteoclasts, then fresh culture medium containing the dye in a 1:10 ratio was added to the well. After 4 h at 37 °C, the fluorescence was read at 530ex-590em nm wavelengths by a Micro Plate reader (VICTOR X2030, Perkin Elmer, Milano, Italy) and expressed as relative fluorescence units (RFU). Since the scaffolds have a composition and a porous structure which tends to adsorb liquids, and thus the dye, a further 3 h incubation with growth medium only was performed to allow the release of the retained reagent. The values of fluorescence read after the second incubation were then added to the previous obtained results. The simple mixture of culture medium and reagent was read and subtracted to correct for the background fluorescence.

### 2.6 Gene expression analysis

Total RNA was isolated from MSCs grown on the scaffolds and separately from osteoclasts grown on the bottom of wells after 7 and 14 days in co-culture. The expression of Runt-related transcription factor 2 (*RUNX2*), Transcription factor Sp7 (*SP7*), Alkaline Phosphatase (*ALPL*), Osteonectin (*SPARC*), Osteopontin (*SPP1*), Osteocalcin (*BGLAP*), type 1 collagen (*COL1A1*), Caspase 3 (*CASP3*), Osteoprotegerin (*OPG*), Receptor activator of nuclear factor kappa-*Β* ligand (*RANKL*), and Vascular Endothelial Growth Factor A (*VEGFA*) for MSCs was assessed. For osteoclast, the expression of Osteoclast associated, Ig-like receptor (*OSCAR*), Cathepsin K (*CTSK*), and Acid Phosphatase 5 Tartrate Resistant (*ACP5*) was evaluated. Briefly, 1 mL of TRIzol reagent (Ambion, Life Technologies, Carlsbad, CA) was added to each sample and incubated for 5 min at room temperature. Chloroform was then added in a 1:5 ratio, and samples were centrifugated at 12.000 RCF at 4 °C for 15 min, after which the aqueous phase was collected, and an equal volume of cold 75% ethanol was added. Finally, purification step was performed using the Purelink™ RNA miniKit (Ambion, Life Technologies, Carlsbad, CA) following the manufacturer’s instructions. The RNA was then quantified by a spectrophotometer (NANODROP 2000, Thermo Scientific) and reverse transcribed using the Superscript Vilo cDNA synthesis kit (Life Technologies). Each sample was diluted to a final concentration of 5 ng/μl, and 10 ng of cDNA were tested in duplicate for each sample. Gene expression was evaluated by semiquantitative Real Time PCR analysis (qPCR) using the SYBR green PCR kit (Qiagen GmbH, Hilden, Germany) in a Light Cycler 2.0 Instrument (Roche Diagnostics, GmbH, Manheim, Germany). The protocol was structured as follows: denaturation cycle at 95 °C for 15 min, amplification (95 °C for 15 s, appropriate annealing temperature for each target for 20 s, and 72 °C for 20 s) for 25 to 40 cycles, and melting curve analysis to check for amplicon specificity. The mean threshold cycle was determined for each sample and used for the calculation of relative expression using the Livak method (2^-ΔΔCt^) (26), with glyceraldehyde-3-phosphate dehydrogenase (*GAPDH*) as the reference gene and the OC scaffold without modifications as calibrator. More detail on the evaluated markers, primers, and protocols are shown in Table 1.

**Table 1:** Sequence of primers, amplicon length, and annealing temperatures used for the evaluation of gene expression by qPCR.

GENE	Primer forward	Primer reverse	Amplicon length	Annealing temperature
*ACP5*	5’-GAAGCGCAGATAGCCGTT-3’	5’-GGTCACTGCCTACCTGTG-3’	148 bp	60 °C
*ALPL*	QuantiTect Primer Assay (Qiagen) Hs_ALPL_1_SG		110 bp	55 °C
*BGLAP*	QuantiTect Primer Assay (Qiagen) Hs_BGLAP_1_SG		90 bp	55 °C
*CASP3*	QuantiTect Primer Assay (Qiagen) Hs_CASP3_1_SG		147 bp	55 °C
*COL1A1*	QuantiTect Primer Assay (Qiagen) Hs_COL1A1_1_SG		118 bp	55 °C
*CTSK*	5’-CAGACAACAGATTTCCATCAGC-3’	5’-CTTCTTCCATAGCTCCCAGTG-3’	118 bp	60 °C
*GAPDH*	QuantiTect Primer Assay (Qiagen) Hs_GAPDH_1_SG		95 bp	55 °C
*OPG*	QuantiTect Primer Assay (Qiagen) Hs_TNFRSF11B_1_SG		107 bp	55 °C
*OSCAR*	QuantiTect Primer Assay (Qiagen) Hs_OSCAR_1_SG		137 bp	55 °C
*SP7*	QuantiTect Primer Assay (Qiagen) Hs_SP7_1_SG		120 bp	55 °C
*RANKL*	QuantiTect Primer Assay (Qiagen) Hs_TNFSF11_1_SG		91 bp	55 °C
*RUNX2*	QuantiTect Primer Assay (Qiagen) Hs_RUNX2_1_SG		101 bp	55 °C
*SPARC*	QuantiTect Primer Assay (Qiagen) Hs_SPARC_1_SG		60 bp	55 °C
*SPP1*	QuantiTect Primer Assay (Qiagen) Hs_SPP1_1_SG		115 bp	55 °C
*VEGFA*	QuantiTect Primer Assay (Qiagen) Hs_VEGFA_6_SG		99 bp	55 °C

### 2.7 Statistical analysis

Statistical analyses were performed with GraphPad Prism software 9.5.1. Data are reported as mean ± standard deviations (SD) at a significance level of *p* < 0.05. After having verified normal distribution and homogeneity of variance, a two-way ANOVA was done, followed by Dunnett’s test to detect the significant differences among the modified biomaterials and OC at each timepoint, while Holm-Sidak’s test was performed to detect the significant differences among experimental times for the same scaffold.

## 3 Results

### 3.1 Effects of the modified scaffolds on MSCs’ morphology

**Figure 1:**
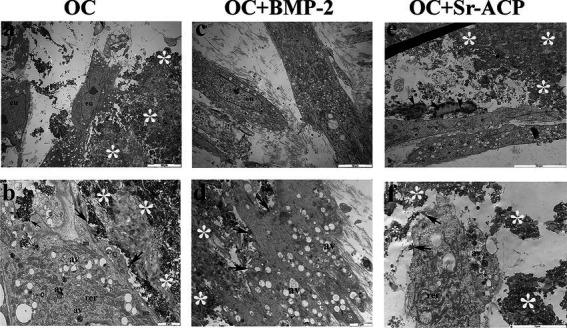
Morphology of MSCs grown on the different scaffolds. Representative TEM images of MSC/scaffold interactions. AV, autophagic vacuoles; black arrow, focal contact; black arrowhead, chromatin condensation/margination; EU, euchromatin; M, mitochondria; RER, rough endoplasmic reticulum; white asterisk, scaffold. **(a,c,e)** Scale bar: 10 μm; **(b,d)** Scale bar: 2 μm; **(f)** Scale bar: 5 μm.

**Figure 2:**
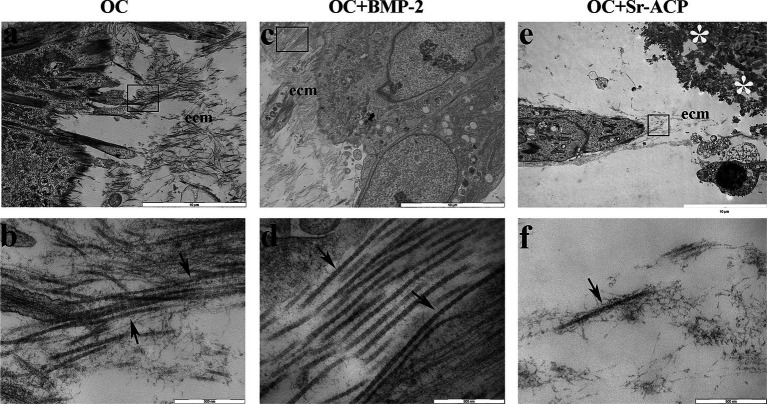
Matrix secretion by MSCs grown on the different scaffolds. Representative TEM images of ECM deposition after 14 days of culture. Black arrow, banded collagen fibers; ECM, extracellular matrix; white asterisk, scaffold. The lower panel represents a higher magnification of the area indicated by black boxes in the upper panel. **(a,c,e)** Scale bar: 10 μm; **(b,d,f)** scale bar: 500 nm.

MSCs cultured on scaffolds presented a good cellular ultrastructure in terms of abundant rough endoplasmic reticulum (rer), well-preserved mitochondria (m), and elongated nuclei with highly dispersed euchromatin (eu) on TEM after 14 days, with no evident differences among all scaffolds (Figures 1A,C). Numerous autophagic vacuoles (av) were also present inside the cytoplasm. Several focal contacts (arrows) were also visible between the cell membrane and HA integrated into the scaffold surfaces or Sr-ACP granules surface (Figures 1B,D,F). The cells grown on the OC + Sr-ACP biomaterial exhibited features for secondary necrosis characterized by rupture of cytoplasmic membrane, swelling, and chromatin margination (Figure 1E). In addition, the cells seeded on all three scaffolds secreted extracellular matrix components (ecm) characterized by banded collagen fibers with different spatial organization (Figures 2A,C,E). In particular, the fibers appeared more abundant and better organized in parallel bundles in the OC + BMP-2 scaffold compared to the other scaffolds (Figures 2B,D,F).

### 3.2 Effects of the modified scaffolds on MSCs’ metabolic activity

**Figure 3:**
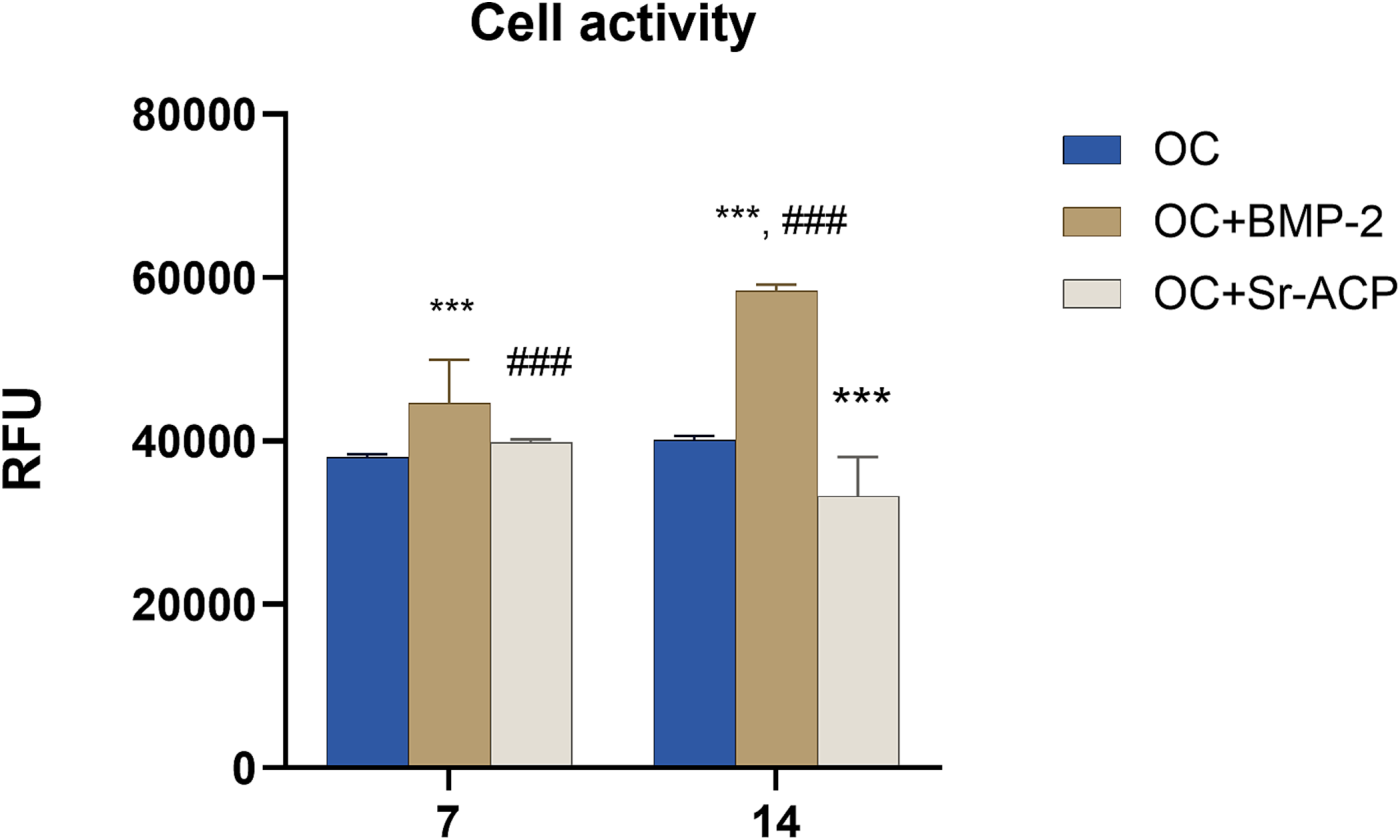
Cell activity of MSCs grown on the different scaffolds. The results were evaluated by Alamar Blue assay after 7 and 14 days of culture and expressed as relative fluorescent units (RFU). Comparisons: ***p < 0.001 vs. OC; ###*p* < 0.001 vs. day 7. Mean ± SD, *n* = 3 duplicates.

The MSCs’ metabolic activity, evaluated by the Alamar Blue assay, was significantly higher in the presence of the OC + BMP-2 scaffold at both 7 and 14 days compared to the control scaffold (117.3 and 145.45%, respectively), with a significant time-dependent increase (130.9%) (Figure 3). No increase was observed in the control scaffold. Conversely, the cell activity in the presence of the OC + Sr-ACP scaffold at 14 days was significantly lower than with the control scaffold (82.9%), with a time-dependent decrease (83.5%) (Figure 3).

### 3.3 Effects of the modified scaffolds on MSCs’ gene expression

**Figure 4:**
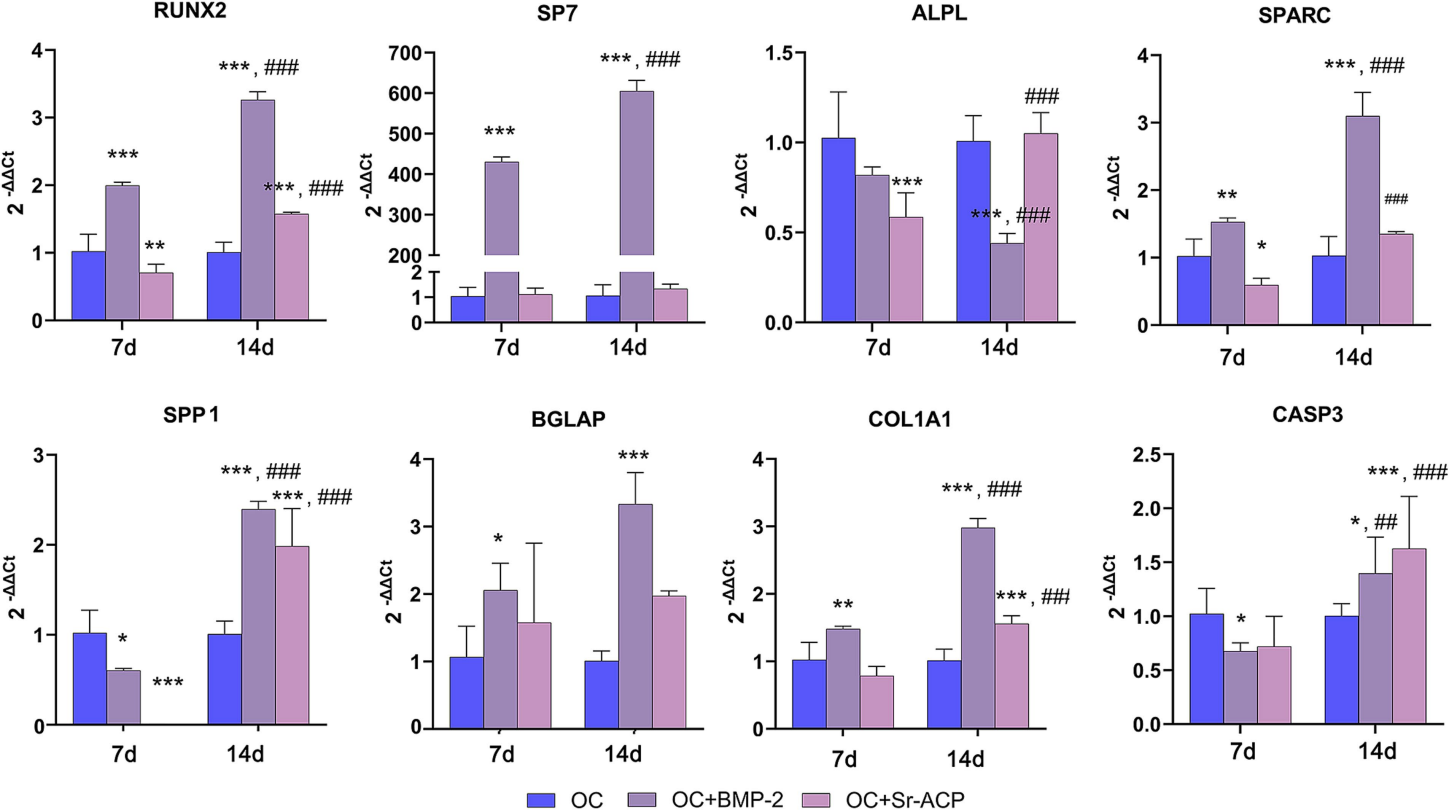
Expression of genes involved in differentiation and apoptosis of MSCs cultured on scaffolds. *RUNX2*, *SP7*, *ALPL*, *SPARC*, *SPP1*, *BGLAP*, *COL1A1*, and *CASP3* levels after 7 and 14 days of culture on OC, OC + BMP-2, and OC + Sr-ACP scaffolds. The results are normalized to *GAPDH* and expressed as 2^-ΔΔCt^ fold change relative to the reference group (OC), considered as 1, at each timepoint. Comparisons: *p < 0.05, **p < 0.005, ***p < 0.001 vs. OC at each timepoint, ^##^*p* < 0.005, ^###^*p* < 0.001 vs. 7 days. Mean ± SD, *n* = 3 duplicates.

The mRNA levels for the *RUNX2, SP7, SPARC*, and *COL1A1* in cells on the OC + BMP-2 scaffold were significantly higher both at 7 and 14 days compared to the control scaffold. On the contrary, their expression in cells grown on the OC + Sr-ACP scaffold was significantly lower vs. the control scaffold at 7 days, except for *SP7* and *COL1A1,* the latter reaching higher levels at 14 days. A similar trend was observed for *BGLAP*, whereas a different result was observed with *ALPL*: its expression on the OC + BMP-2 scaffold was significantly lower than on the OC scaffold after 14 days, following a decreasing trend between the first and the second weeks, while on the OC + Sr-ACP scaffold was significantly lower compared to the OC scaffold after 7 days, but it increased at 14 days. Finally, the expression of *SPP1* was induced by the OC + BMP-2 scaffold and by the OC + Sr-ACP scaffold at 14 days (Figure 4).

The MSCs’ apoptosis was assessed by evaluating the mRNA levels for *CASP3*, which were significantly lower in the presence of the OC + BMP-2 scaffolds with respect to the control at 7 days. At 14 days, on the contrary, a significant time-dependent increase was observed for both modified scaffolds, reaching significantly higher levels than controls (Figure 4).

### 3.4 Effects of the modified scaffolds on MSCs’ interaction with endothelial cells and osteoclasts

**Figure 5:**
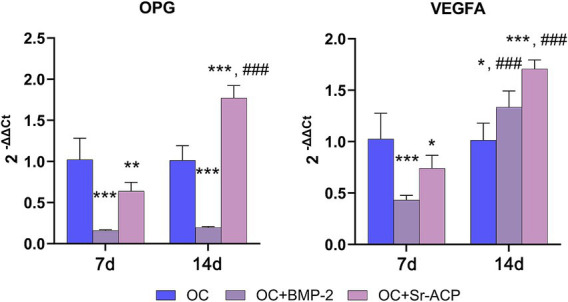
Expression of genes involved in angiogenesis and osteoclasts induction in MSCs cultured on scaffolds. mRNA levels for *OPG* and *VEGFA* after 7 and 14 days of MSCs culture on OC, OC + BMP-2, and OC + Sr-ACP scaffolds. The results are normalized to *GAPDH* and expressed as 2^-ΔΔCt^ fold change relative to the reference group (OC), considered as 1, at each timepoint. Comparisons: *p < 0.05, **p < 0.005, ***p < 0.001 vs. OC at each timepoint, ^###^p < 0.001 vs. 7 days. Mean ± SD, *n* = 3 duplicates.

As the MSCs were grown in a co-culture system in the presence of osteoclasts and conditioned medium of endothelial cells, the expression of *OPG* and *RANKL*, related to the osteoclasts’ activity, and the angiogenic marker *VEGFA*, was also evaluated in MSCs. The expression of *VEGFA* was lower in the presence of both modified scaffolds than with the OC scaffold at 7 days. In both cases, its expression reached significantly higher levels than the control at 14 days (Figure 5). After 7 days of co-culture, the *OPG* expression by MSCs’ grown on both modified biomaterials was significantly lower than in the presence of the unmodified. The result was confirmed for the OC + BMP-2 scaffold at 14 days, whereas with the OC + Sr-ACP scaffold its expression reached a significantly higher level than the control scaffold at 14 days (Figure 5). *RANKL* expression, however, was undetectable in these cells in all the experimental conditions (data not shown).

### 3.5 Effects of the modified scaffolds on osteoclasts

**Figure 6:**
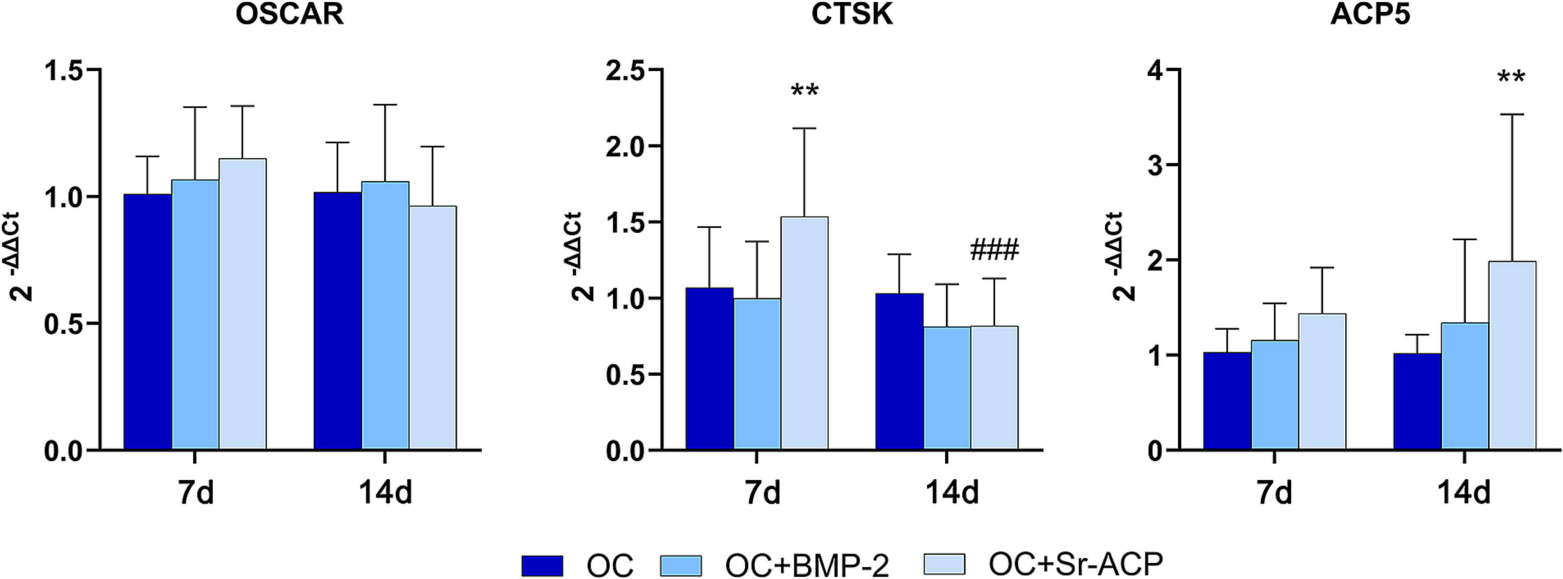
Expression of genes related to osteoclasts. mRNA levels for *OSCAR*, *CTSK*, and *ACP5* after 7 and 14 days of co-culture with MSCs. The results are normalized to *GAPDH* and expressed as 2^-ΔΔCt^ fold change relative to the reference group (OC), considered as 1, at each timepoint. Comparisons: **p < 0.005 vs. OC. ###*p* < 0.001 vs. 7 days. Mean ± SD, *n* = 3 duplicates.

The presence of the OC + BMP-2 scaffold did not induce the expression of the osteoclasts’ genes *OSCAR, CTSK*, and *ACP5*. In fact, no differences were observed with respect to the control scaffold, and no increase over time was seen for any of them. On the other hand, the expression of *CTSK* and *ACP5* was significantly higher than control with the OC + Sr-ACP scaffold at day 7 and day 14, respectively, with a significant decrease at day 14 for *CTSK* (Figure 6).

## 4 Discussion

The scaffold employed in this study corresponds to the subchondral bone–like layer of one of the most widely used implants for the treatment of osteochondral lesions (OCL). Despite its favorable clinical outcomes, this implant still fails to achieve a more complete subchondral bone regeneration (5, 12).

To address this limitation, two strategies were proposed, both aimed at enhancing the osteoinductive potential of this scaffold layer. These approaches are based on the well-documented osteogenic activity of BMP-2 (27), which has already demonstrated efficacy in combination with various biomaterials (28) and through a calcium phosphate and ion-rich microenvironment as provided by the association of ACP with Sr (18, 29, 30).

The main finding of this study is that both osteochondral scaffold augmentation strategies effectively supported MSC adhesion and proliferation. In particular, the BMP-2–modified scaffold promoted MSC differentiation toward a more mature osteoblastic phenotype, whereas the Sr-ACP–modified scaffold appeared to be more prone to inducing osteoclast activation.

A preliminary analysis of the interaction between MSCs and the scaffold surfaces, performed using high-resolution transmission electron microscopy, demonstrated that all scaffolds supported MSC adhesion, maintaining an overall healthy cellular status. This was evidenced by the well-preserved morphology of mitochondria, endoplasmic reticulum, and nuclei with dispersed chromatin. Previous studies have shown that the surface characteristics of scaffolds can influence the morphology, behavior, and differentiation of bone marrow–derived cells (31). Notably, some differences in cellular behavior were observed between the two modified biomaterials. Cells cultured on the BMP-2–loaded scaffold exhibited increased extracellular matrix (ECM) production, indicative of intense metabolic activity, in agreement with the results of the Alamar Blue assay and consistent with differentiation toward a more mature phenotype. Accordingly, numerous cytoplasmic autophagic vacuoles were detected in cells on this scaffold, suggesting an increased energy demand by MSCs during matrix synthesis (32).

The expression levels of most of the analyzed markers, including *RUNX2*, *SP7*, *SPARC*, *SSP1*, *BGLAP*, and *COL1A1*, indicated that the presence of BMP-2 promoted MSC differentiation. All these genes are known to play key roles in the regulation of the osteogenic process (33–37), encoding proteins involved in bone matrix organization and turnover (38, 39), or participating in the complex signaling interactions among different bone cell types, such as osteoblasts and osteoclasts (25).

Overall, their expression was more markedly upregulated in the presence of the BMP-2-loaded scaffold than with the Sr-ACP scaffold, as could be expected given that BMPs have long been recognized as the most potent osteogenic factors (40). Xu et al. demonstrated that the Col–Mg–HA layer is capable of retaining and gradually releasing BMP-2, suggesting that a direct interaction between the material and the cells may represent the main mechanism through which this growth factor exerts its biological effects (17). In contrast, *ALPL* expression exhibited a different trend, showing lower levels than the normal scaffold and a progressive decrease over time in the presence of BMP-2, whereas the Sr-ACP scaffold induced a time-dependent increase, reaching significantly higher values than the normal scaffold after 14 days. In the current literature, there is no agreement regarding the response of MSCs to biomaterials in terms of *ALPL* activity, as some authors have reported a well-defined peak at day 7 followed by a decline (41), whereas others have observed maximum activity at day 14 (42). In the present study, considering the differential expression of the *ALPL* gene together with that of late differentiation markers, the most plausible scenario is that MSCs underwent maturation and differentiation more rapidly on the BMP-2-loaded scaffold (upregulation of late genes accompanied by a decrease in *ALPL*), whereas on the Sr-ACP scaffold the process occurred more slowly (lower expression of late genes and an increase in *ALPL*). These considerations suggest that cells cultured on the biomaterial enriched with calcium phosphate and strontium were less active in terms of differentiation and matrix production. Supporting this hypothesis, ultra-high magnification imaging of MSCs grown on the Sr-ACP–modified scaffold revealed apoptotic nuclei characterized by typical chromatin condensation/margination. This phenotype was further confirmed by the assessment of cell activity and *CASP3* mRNA expression, which were significantly lower and higher, respectively, than those observed in the control scaffold after 14 days. Previous studies have reported that elevated strontium concentrations can compromise the viability of adipose-derived stem cells, inducing apoptosis-related features (43), whereas other authors have reported opposite findings (44).

However, an increase in *CASP3* was observed over time in both materials, more markedly on Sr-ACP. The mentioned phenomenon could reflect the different roles of this enzyme, as described by Ghani et al.: *CASP3* up-regulation does not necessarily indicate extensive apoptosis, as caspase activation can participate in non-lethal processes of stem cell differentiation such as cytoskeletal remodeling and osteogenic maturation. The apoptotic features observed predominantly in the Sr-ACP group therefore likely reflect stress-induced apoptosis, rather than the physiological caspase activation seen during differentiation of MSCs in presence of BMP-2 (45).

The Sr-ACP–containing scaffolds used in this study (Sr content in Sr-ACP = 2.49 wt%, a relatively low amount) have been tested previously, characterized and classified as non-cytotoxic by Xu et al. (18). Indeed, the numerous focal adhesions observed under all conditions, including those with Sr-ACP granules, further confirmed a good cell–scaffold interaction, consistent with previous findings (16). Biomaterial properties—including porosity, surface structure, and chemical composition—have been shown to modulate the host cell response (46), while the secretome of apoptotic metabolites can elicit signals that promote cell proliferation, reduce and suppress inflammation in murine arthritis models (47). In particular, it has been reported that MSCs cultured on calcium phosphate-based biomaterials may undergo apoptosis, thereby shifting their paracrine secretion profile toward the release of osteoclastogenic factors (48).

The expression of specific osteoclastic markers, such as CTSK and ACP5, in the co-culture system was enhanced in the presence of the Sr-ACP–modified scaffold, reaching significantly higher levels than those observed under the other two conditions. In contrast, no differences in the expression of these markers were detected between the unmodified and BMP-2–loaded scaffolds. Together, these data suggest that the incorporation of Sr-ACP granules within the scaffold may promote osteoclast activity both directly by osteoclasts themselves and indirectly through MSC-mediated mechanisms, in a manner distinct from the BMP-2–loaded biomaterial. In addition to their well-established role in scaffold resorption (49), osteoclasts also contribute to bone matrix maturation during intramembranous ossification (50); thus, their activation could further enhance the bone formation process.

A significantly higher OPG expression by MSCs was observed on the Sr-ACP scaffold compared with the normal one, consistent with previously reported findings about osteoblasts, where Sr. ions were shown to increase OPG and to decrease RANK (51). Although this observation appears to contrast with the enhanced osteoclastic activity detected on the same scaffolds—given that OPG typically inhibits, rather than promotes, osteoclast activation—it may instead suggest a balanced regulatory mechanism between osteogenesis and bone resorption, orchestrated by MSCs. Nevertheless, calcium phosphate–based ACP granules have been shown to effectively modulate osteoclast differentiation, thereby promoting osteoclast-mediated osseointegration between the material and the surrounding bone tissue (52, 53); thus, their activation within this environment could be expected.

Finally, *VEGFA* expression by MSCs increased over time with both modified biomaterials, reaching higher levels than those observed with the unmodified scaffold, particularly in the case of the Sr-ACP scaffolds. Strontium ions have been reported to directly stimulate *VEGFA* production (54, 55), including when combined with calcium and hydroxyapatite (HA) (56). *VEGFA* plays a dual role in osseointegration, as it promotes angiogenesis and exerts autocrine/paracrine effects on the maturation of osteoblast-like cells (57). Moreover, it can enhance bone formation not only by promoting vascularization, but also by directly influencing osteogenesis through the recruitment of osteoblasts and osteoclasts (58), in agreement with the present findings indicating an increased regenerative potential.

Finally, to speculate on the role of Sr., a very interesting paper of Li et al. observed a positive role of this ion, when associated with ceramic biomaterials and magnesium, on both osteogenesis and chondrogenesis, as well as on improving the inflammatory microenvironment. This multifaceted role of Sr. would deserve to be further explored in the context of osteochondral regeneration (59).

This study presents certain limitations. Although a co-culture system involving three distinct cell types was employed to investigate cell–cell interactions, the *in vitro* static model represents a simplified system and cannot fully reproduce the *in vivo* microenvironment. More physiologically relevant approaches could involve perfusion cultures using bioreactor systems, which better mimic the mechanical stresses occurring *in vivo* enhancing cellular osteogenesis and mineralization (60). Furthermore, only three specific genes were analyzed to assess osteoclast behavior.

Additional investigations focused on assays typical of the cells involved in this culture model could further strengthen the results: ALP activity, release of matrix proteins and calcium deposition by the osteoblasts, as well as TRAP activity and resorption pits by the osteoclasts.

Since BMP-2, ACP, and Sr. are well known and included in various scaffolds, and the reference material is already in clinical use, this work was designed as ultrastructural observation and mechanistic study at the gene level to observe which mechanisms were most modified by BMP-2 and which by Sr-ACP when associated to the Col/Col-Mg-HA scaffold. This frame should be then complemented by functional tests in a future study.

Nevertheless, despite these limitations, the present results demonstrate the suitability of both scaffold modification strategies to effectively support bone regeneration processes.

## 5 Conclusion

In conclusion, both strategies employed to improve the osteoinductive potential of the subchondral bone–like layer of this Col/Col-Mg-HA scaffold effectively supported adhesion, growth, and differentiation of human MSCs. The co-culture system highlighted how the BMP-2-based strategy promoted MSC differentiation toward a more mature osteogenic phenotype *in vitro*, whereas the Sr-ACP strategy primarily favored osteoclast activation which may facilitate scaffold resorption *in vivo*. These findings open new possible scenarios, such as the investigation of the effectiveness of the scaffold modified with the combined augmentation strategies. The evaluation of these results still needs to be confirmed in appropriate translational models.

## Data Availability

The raw data supporting the conclusions of this article will be made available by the authors, without undue reservation.

## References

[1] Lepage SIM Robson N Gilmore H Davis O Hooper A St John S . Beyond cartilage repair: the role of the osteochondral unit in joint health and disease. Tissue Eng Part B Rev. (2019) 25:114–25. doi: 10.1089/ten.TEB.2018.0122, 30638141 PMC6486663

[2] Wei W Dai H. Articular cartilage and osteochondral tissue engineering techniques: recent advances and challenges. Bioact Mater. (2021) 6:4830–55. doi: 10.1016/j.bioactmat.2021.05.011, 34136726 PMC8175243

[3] Solheim E Krokeide AM Melteig P Larsen A Strand T Brittberg M. Symptoms and function in patients with articular cartilage lesions in 1,000 knee arthroscopies. Knee Surg Sports Traumatol Arthrosc. (2016) 24:1610–6. doi: 10.1007/s00167-014-3472-9 25502829

[4] De Marziani L Boffa A Di Martino A Andriolo L Reale D Bernasconi A . The reimbursement system can influence the treatment choice and favor joint replacement versus other less invasive solutions in patients affected by osteoarthritis. J Exp Orthop. (2023) 10:146. doi: 10.1186/s40634-023-00699-5, 38135778 PMC10746689

[5] Di Martino A Perdisa F Filardo G Busacca M Kon E Marcacci M . Cell-free biomimetic osteochondral scaffold for the treatment of knee lesions: clinical and imaging results at 10-year follow-up. Am J Sports Med. (2021) 49:2645–50. doi: 10.1177/03635465211029292, 34283948

[6] Perdisa F Bordini B Salerno M Traina F Zaffagnini S Filardo G. Total knee arthroplasty (TKA): when do the risks of TKA overcome the benefits? Double risk of failure in patients up to 65 years old. Cartilage. (2023) 14:305–11. doi: 10.1177/19476035231164733, 37073516 PMC10601565

[7] GBD 2021 Osteoarthritis Collaborators (2023). Global, regional, and national burden of osteoarthritis, 1990-2020 and projections to 2050: a systematic analysis for the global burden of disease study 2021. 5:e508-e522. doi: 10.1016/S2665-9913(23)00163-7 PMC10477960 37675071

[8] Nham FH Patel I Zalikha AK El-Othmani MM. Epidemiology of primary and revision total knee arthroplasty: analysis of demographics, comorbidities and outcomes from the national inpatient sample. Art. (2023) 5:18. doi: 10.1186/s42836-023-00175-6 PMC10068145 37004093

[9] Serre CM Papillard M Chavassieux P Voegel JC Boivin G. Influence of magnesium substitution on a collagen-apatite biomaterial on the production of a calcifying matrix by human osteoblasts. J Biomed Mater Res. (1998) 42:626–33. doi: 10.1002/(sici)1097-4636(19981215)42:4<>3.0.co;2-s, 9827688

[10] Kon E Filardo G Robinson D Eisman JA Levy A Zaslav K . Osteochondral regeneration using a novel aragonite-hyaluronate bi-phasic scaffold in a goat model. Knee Surg Sports Traumatol Arthrosc. (2014) 22:1452–64. doi: 10.1007/s00167-013-2467-2, 23479056

[11] Tampieri A Celotti G Landi E Sandri M Roveri N Falini G. Biologically inspired synthesis of bone-like composite: self-assembled collagen fibers/hydroxyapatite nanocrystals. J Biomed Mater Res A. (2003) 67:618–25. doi: 10.1002/jbm.a.10039 14566805

[12] Kon E Filardo G Perdisa F Venieri G Marcacci M. Clinical results of multilayered biomaterials for osteochondral regeneration. J Exp Orthop. (2014) 1:10. doi: 10.1186/s40634-014-0010-0, 26914755 PMC4648845

[13] Zhou H Liang B Jiang H Deng Z Yu K. Magnesium-based biomaterials as emerging agents for bone repair and regeneration: from mechanism to application. J Magnesium Alloys. (2021) 9:779–804. doi: 10.1016/j.jma.2021.03.004

[14] Rutgers M Saris DB Vonk LA van Rijen MH Akrum V Langeveld D . Effect of collagen type I or type II on chondrogenesis by cultured human articular chondrocytes. Tissue Eng Part A. (2013) 19:59–65. doi: 10.1089/ten.TEA.2011.0416, 22861168

[15] Kon E Delcogliano M Filardo G Busacca M Di Martino A Marcacci M. Novel nano-composite multilayered biomaterial for osteochondral regeneration: a pilot clinical trial. Am J Sports Med. (2011) 39:1180–90. doi: 10.1177/0363546510392711, 21310939

[16] Pagani S Salerno M Filardo G Locs J van Osch GJVM Vecstaudza J . Human osteoblasts’ response to biomaterials for subchondral bone regeneration in standard and aggressive environments. Int J Mol Sci. (2023) 24:14764. doi: 10.3390/ijms241914764, 37834212 PMC10573262

[17] Xu J Fahmy-Garcia S Wesdorp MA Kops N Forte L De Luca C . Effectiveness of BMP-2 and PDGF-BB adsorption onto a collagen/collagen-magnesium-hydroxyapatite scaffold in weight-bearing and non-weight-bearing osteochondral defect bone repair: in vitro, ex vivo and in vivo evaluation. J Funct Biomater. (2023) 14:111. doi: 10.3390/jfb14020111, 36826910 PMC9961206

[18] Xu J Vecstaudza J Wesdorp MA Labberté M Kops N Salerno M . Incorporating strontium enriched amorphous calcium phosphate granules in collagen/collagen-magnesium-hydroxyapatite osteochondral scaffolds improves subchondral bone repair. Mater Today Bio. (2024) 25:100959. doi: 10.1016/j.mtbio.2024.100959, 38327976 PMC10847994

[19] Pagani S Salerno M Locs J Vecstaudza J Dolcini L Fini M . Enhanced osteogenic response to an osteochondral scaffold modified with BMP-2 or strontium-enriched amorphous calcium phosphate in a co-culture in vitro model. J Funct Biomater. (2025) 16:302. doi: 10.3390/jfb16080302 40863322 PMC12387257

[20] Pagani S Torricelli P Veronesi F Salamanna F Cepollaro S Fini M. An advanced tri-culture model to evaluate the dynamic interplay among osteoblasts, osteoclasts, and endothelial cells. J Cell Physiol. (2018) 233:291–301. doi: 10.1002/jcp.25875, 28240358

[21] Grassel S Ahmed N. Influence of cellular microenvironment and paracrine signals on chondrogenic differentiation. Front Biosci. (2007) 12:4946–56. doi: 10.2741/2440 17569622

[22] Muenzebrock KA Kersten V Alblas J Garcia JP Creemers LB. The added value of the ‘co’ in co-culture systems in research on osteoarthritis pathology and treatment development. Front Bioeng Biotechnol. (2022) 10:843056. doi: 10.3389/fbioe.2022.843056, 35309991 PMC8927651

[23] Portal-Núñez S Lozano D Esbrit P. Role of angiogenesis on bone formation. Histol Histopathol. (2012) 27:559–66. doi: 10.14670/HH-27.559, 22419020

[24] Burger MG Grosso A Briquez PS Born GME Lunger A Schrenk F . Robust coupling of angiogenesis and osteogenesis by VEGF-decorated matrices for bone regeneration. Acta Biomater. (2022) 149:111–25. doi: 10.1016/j.actbio.2022.07.014, 35835287

[25] Hayami T Kapila YL Kapila S. Divergent upstream osteogenic events contribute to the differential modulation of MG63 cell osteoblast differentiation by MMP-1 (collagenase-1) and MMP-13 (collagenase-3). Matrix Biol. (2011) 30:281–9. doi: 10.1016/j.matbio.2011.04.003, 21539914 PMC3116144

[26] Livak KJ Schmittgen TD. Analysis of relative gene expression data using real-time quantitative PCR and the 2(-Delta Delta C(T)) method. Methods. (2001) 25:402–8. doi: 10.1006/meth.2001.1262 11846609

[27] Halloran D Durbano HW Nohe A. Bone morphogenetic protein-2 in development and bone homeostasis. J Dev Biol. (2020) 8:19. doi: 10.3390/jdb8030019, 32933207 PMC7557435

[28] Kim SE Yun YP Lee JY Shim JS Park K Huh JB. Co-delivery of platelet-derived growth factor (PDGF-BB) and bone morphogenic protein (BMP-2) coated onto heparinized titanium for improving osteoblast function and osteointegration. J Tissue Eng Regen Med. (2015) 9:E219–28. doi: 10.1002/term.1668 23288808

[29] Capuccini C Torricelli P Boanini E Gazzano M Giardino R Bigi A. Interaction of Sr-doped hydroxyapatite nanocrystals with osteoclast and osteoblast-like cells. J Biomed Mater Res A. (2009) 89:594–600. doi: 10.1002/jbm.a.31975, 18437694

[30] Schumacher M Gelinsky M. Strontium modified calcium phosphate cements - approaches towards targeted stimulation of bone turnover. J Mater Chem B. (2015) 3:4626–40. doi: 10.1039/c5tb00654f, 32262477

[31] Bajcsy P Yoon S Florczyk SJ Hotaling NA Simon M Szczypinski PM . Modeling, validation and verification of three-dimensional cell-scaffold contacts from terabyte-sized images. BMC Bioinformatics. (2017) 18:526. doi: 10.1186/s12859-017-1928-x, 29183290 PMC5706418

[32] Nuschke A Rodrigues M Stolz DB Chu CT Griffith L Wells A. Human mesenchymal stem cells/multipotent stromal cells consume accumulated autophagosomes early in differentiation. Stem Cell Res Ther. (2014) 5:140. doi: 10.1186/scrt530, 25523618 PMC4446103

[33] Chan WCW Tan Z To MKT Chan D. Regulation and role of transcription factors in osteogenesis. Int J Mol Sci. (2021) 22:5445. doi: 10.3390/ijms22115445 34064134 PMC8196788

[34] Chen Q Shou P Zhang L Xu C Zheng C Han Y . An osteopontin-integrin interaction plays a critical role in directing adipogenesis and osteogenesis by mesenchymal stem cells. Stem Cells. (2014) 32:327–37. doi: 10.1002/stem.1567, 24123709 PMC3961005

[35] Chen XJ Shen YS He MC Yang F Yang P Pang FX . Polydatin promotes the osteogenic differentiation of human bone mesenchymal stem cells by activating the BMP2-Wnt/β-catenin signaling pathway. Biomed Pharmacother. (2019) 112:108746. doi: 10.1016/j.biopha.2019.108746, 30970530

[36] Gomathi K Akshaya N Srinaath N Moorthi A Selvamurugan N. Regulation of Runx2 by post-translational modifications in osteoblast differentiation. Life Sci. (2020) 245:117389. doi: 10.1016/j.lfs.2020.117389, 32007573

[37] Liu Q Li M Wang S Xiao Z Xiong Y Wang G. Recent advances of Osterix transcription factor in osteoblast differentiation and bone formation. Front Cell Dev Biol. (2020) 8:601224. doi: 10.3389/fcell.2020.601224 33384998 PMC7769847

[38] Amirrah IN Lokanathan Y Zulkiflee I Wee MFMR Motta A Fauzi MB. A comprehensive review on collagen type I development of biomaterials for tissue engineering: from biosynthesis to bioscaffold. Biomedicine. (2022) 10:2307. doi: 10.3390/biomedicines10092307, 36140407 PMC9496548

[39] Zhu YS Gu Y Jiang C Chen L. Osteonectin regulates the extracellular matrix mineralization of osteoblasts through P38 signaling pathway. J Cell Physiol. (2020) 235:2220–31. doi: 10.1002/jcp.29131, 31489629

[40] Luu HH Song WX Luo X Manning D Luo J Deng ZL . Distinct roles of bone morphogenetic proteins in osteogenic differentiation of mesenchymal stem cells. J Orthop Res. (2007) 25:665–77. doi: 10.1002/jor.20359 17290432

[41] Chen M Le DQS Kjems J Bünger C Lysdahl H. Improvement of distribution and osteogenic differentiation of human mesenchymal stem cells by hyaluronic acid and β-tricalcium phosphate-coated polymeric scaffold in vitro. Biores Open Access. (2015) 4:363–73. doi: 10.1089/biores.2015.0021, 26487981 PMC4599126

[42] Jensen J Kraft DCE Lysdahl H Foldager CB Chen M Kristiansen AA . Functionalization of polycaprolactone scaffolds with hyaluronic acid and β-TCP facilitates migration and osteogenic differentiation of human dental pulp stem cells in vitro. Tissue Eng Part A. (2015) 21:729–39. doi: 10.1089/ten.TEA.2014.0177, 25252795 PMC4334472

[43] Aimaiti A Maimaitiyiming A Boyong X Aji K Li C Cui L. Low-dose strontium stimulates osteogenesis but high-dose doses cause apoptosis in human adipose-derived stem cells via regulation of the ERK1/2 signaling pathway. Stem Cell Res Ther. (2017) 8:282. doi: 10.1186/s13287-017-0726-8, 29254499 PMC5735894

[44] Li Y Li J Zhu S Luo E Feng G Chen Q . Effects of strontium on proliferation and differentiation of rat bone marrow mesenchymal stem cells. Biochem Biophys Res Commun. (2012) 418:725–30. doi: 10.1016/j.bbrc.2012.01.088, 22306818

[45] Abdul-Ghani M Megeney LA. Rehabilitation of a contract killer: caspase-3 directs stem cell differentiation. Cell Stem Cell. (2008) 2:515–6. doi: 10.1016/j.stem.2008.05.013, 18522841

[46] Kim H Kumbar SG Nukavarapu SP. Biomaterial-directed cell behavior for tissue engineering. Curr Opin Biomed Eng. (2021) 17:100260. doi: 10.1016/j.cobme.2020.100260, 33521410 PMC7839921

[47] Medina CB Mehrotra P Arandjelovic S Perry JSA Guo Y Morioka S . Metabolites released from apoptotic cells act as tissue messengers. Nature. (2020) 580:130–5. doi: 10.1038/s41586-020-2121-3, 32238926 PMC7217709

[48] Humbert P Brennan MÁ De Lima J Brion R Adrait A Charrier C . Apoptotic mesenchymal stromal cells support osteoclastogenesis while inhibiting multinucleated giant cells formation in vitro. Sci Rep. (2021) 11:12144. doi: 10.1038/s41598-021-91258-4, 34108508 PMC8190145

[49] Detsch R Boccaccini AR. The role of osteoclasts in bone tissue engineering. J Tissue Eng Regen Med. (2015) 9:1133–49. doi: 10.1002/term.1851, 24478169

[50] Nakamura M Aoyama N Yamaguchi S Sasano Y. Expression of tartrate-resistant acid phosphatase and cathepsin K during osteoclast differentiation in developing mouse mandibles. Biomed Res. (2021) 42:13–21. doi: 10.2220/biomedres.42.13, 33563875

[51] Kołodziejska B Stępień N Kolmas J. The influence of strontium on bone tissue metabolism and its application in osteoporosis treatment. Int J Mol Sci. (2021) 22:6564. doi: 10.3390/ijms22126564, 34207344 PMC8235140

[52] Jeong J Kim JH Shim JH Hwang NS Heo CY. Bioactive calcium phosphate materials and applications in bone regeneration. Biomater Res. (2019) 23:4. doi: 10.1186/s40824-018-0149-3 30675377 PMC6332599

[53] Wang X Yu Y Ji L Geng Z Wang J Liu C. Calcium phosphate-based materials regulate osteoclast-mediated osseointegration. Bioact Mater. (2021) 6:4517–30. doi: 10.1016/j.bioactmat.2021.05.003, 34632163 PMC8484898

[54] Sheng X Li C Wang Z Xu Y Sun Y Zhang W . Advanced applications of strontium-containing biomaterials in bone tissue engineering. Mater Today Bio. (2023) 20:100636. doi: 10.1016/j.mtbio.2023.100636 PMC10333686 37441138

[55] Zhu X Kong Y Huang Y Zhao B Wang J. Influence of strontium on vascular endothelial growth factor and fibroblast growth factor 2 expression in rat chondrocytes cultured in vitro. Biol Trace Elem Res. (2019) 190:466–71. doi: 10.1007/s12011-018-1564-y, 30414002

[56] Gu Z Xie H Huang C Peng H Tan H Li L . Effects of strontium-doped calcium polyphosphate on angiogenic growth factors expression of co-culturing system in vitro and of host cell in vivo. RSC Adv. (2014) 4:2783–92. doi: 10.1039/C3RA44323J

[57] Raines AL Berger MB Patel N Hyzy SL Boyan BD Schwartz Z. VEGF-A regulates angiogenesis during osseointegration of Ti implants via paracrine/autocrine regulation of osteoblast response to hierarchical microstructure of the surface. J Biomed Mater Res A. (2019) 107:423–33. doi: 10.1002/jbm.a.36559, 30461195 PMC6892345

[58] Stamnitz S Klimczak A. Mesenchymal stem cells, bioactive factors, and scaffolds in bone repair: from research perspectives to clinical practice. Cells. (2021) 10:1925. doi: 10.3390/cells10081925, 34440694 PMC8392210

[59] Li B Zhang Y Zhou X Wang W Yang F Wei Q Mimicking DOUGONG brackets orchestrate regulating inflammation and mechanical stimuli for osteochondral regeneration using 3D printing. Interdiscip Med 2025. Available online at: https://onlinelibrary.wiley.com/doi/10.1002/inmd.70068 (December 12, 2025)

[60] Zhang ZY Teoh SH Teo EY Khoon Chong MS Shin CW Tien FT . A comparison of bioreactors for culture of fetal mesenchymal stem cells for bone tissue engineering. Biomaterials. (2010) 31:8684–95. doi: 10.1016/j.biomaterials.2010.07.097, 20739062

